# Metabolic syndrome prevalence and its risk factors among adults in China: A nationally representative cross-sectional study

**DOI:** 10.1371/journal.pone.0199293

**Published:** 2018-06-19

**Authors:** Yaru Li, Liyun Zhao, Dongmei Yu, Zhihong Wang, Gangqiang Ding

**Affiliations:** National Institute for Nutrition and Health, Chinese Center for Disease Control and Prevention, Beijing, China; Shanghai Institute of Hypertension, CHINA

## Abstract

**Objectives:**

The aim of this study was to examine sex disparity in metabolic syndrome prevalence and its risk factors among Chinese adults.

**Methods:**

Using the 2010–2012 China National Nutrition and Health Survey (CNNHS), a nationally representative cross-sectional study on nutrition and non-communicable chronic diseases, a total of 98,042 participants aged 18 years and older were included in the analysis. Dietary information was collected with a food frequency questionnaire (FFQ). Metabolic syndrome was defined according to the updated NCEP ATP III criteria. A multivariable logistic regression model was performed to examine the associations between sociodemographic and dietary factors with metabolic syndrome prevalence, and the results are presented using odd ratios (ORs) and 95% confidence intervals (CIs).

**Results:**

The overall standardized prevalence of metabolic syndrome was 24.2% (24.6% in men and 23.8% in women). The metabolic syndrome prevalence was positively associated with age in men and women. The prevalence of metabolic syndrome was negatively associated with the physical activity level among men and inversely associated with the education level among women (*P* for trend < 0.01). Frequent consumption of fungi and algae was an underlying risk factor for metabolic syndrome in men, whereas frequent consumption of nuts and pork was associated with a decreased prevalence of metabolic syndrome in women.

**Conclusions:**

The prevalence of metabolic syndrome in men was not different from that in women. There are sex-specific associations between multiple risk factors and metabolic syndrome.

## Introduction

In 2015, approximately 290 million people had cardiovascular disease (CVD). CVD is the number one cause of mortality in China and accounts for over 40% of total deaths [1]. Metabolic syndrome is characterized by a clustering of CVD risk factors, including abdominal obesity; increased blood pressure, fasting plasma glucose, and triglyceride (TG); and decreased high-density lipoprotein cholesterol (HDL-C) [2]. Exploring the cause of metabolic syndrome prevalence may provide important public health implications for the prevention and management of CVD. The prevalence of metabolic syndrome has increased dramatically worldwide [3–5]. According to the International Collaborative Study of Cardiovascular Disease in ASIA (InterASIA), the age-standardized prevalence of metabolic syndrome was 13.7% among adults aged 35–74 years in China between 2000 and 2001, using the National Cholesterol Education Program Adult Treatment Panel III (NCEP ATP III) criteria [6]. Based on 2010 China Noncommunicable Disease Surveillance data assessed by NCEP ATP III criteria, the prevalence of metabolic syndrome among participants aged 18 years and older was 33.9% [7].

In China, dietary intake has changed substantially, which may be causing the rapidly rising prevalence of metabolic syndrome. Grain intake has decreased significantly, whereas fat intake has increased dramatically. The daily intake of salt is much higher, and the daily intake of vegetables and fruits is lower than recommended [8]. A cross-sectional study explored metabolic syndrome prevalence and associated dietary factors in a sample of urban Chinese adults; however, the dietary intake factors were not fully explored [9]. A prospective study based on the amount of dietary intake showed that the consumption of meat, fried food, and diet soda were adversely associated with incident metabolic syndrome risk, whereas dairy consumption was beneficial [10].

Although growing evidence has suggested that multitudinous factors are associated with metabolic syndrome prevalence [11–13], few studies have investigated the sex disparity associations between risk factors and metabolic syndrome prevalence. Therefore, we aimed to examine sex disparity in metabolic syndrome prevalence and its risk factors among the Chinese population.

## Methods

### Study population

The 2010–2012 China National Nutrition and Health Survey (CNNHS) is a nationally representative cross-sectional study on nutrition and non-communicable chronic diseases. This survey selected 150 survey sites (districts or counties) of 31 provinces, autonomous regions, and municipalities directly under the Chinese central government (excluding Taiwan, Hong Kong, and Macao). The country was divided into four strata according to socioeconomic characteristics: large cities, small-to-medium cities, general rural areas and poor rural areas. The first stage of sampling involved the random selection of 150 survey sites, including 34 survey sites from large cities, 41 survey sites from small-to-medium cities, 45 survey sites from general rural areas, and 30 survey sites from poor rural areas. The second stage involved the random selection of six residential committees (urban) or villages (rural). In the third sampling stage, according to the geographical location of the household, a total of 25 households is considered as a sampling unit. Three sampling units (75 households) were randomly selected from each of the residential committees or villages. In addition, participants from the first sampling unit (25 households) completed an interview with a structured food frequency questionnaire (FFQ).

Individuals with missing data on metabolic syndrome components including waist circumference, TG, HDL-C, blood pressure, and fasting plasma glucose were excluded. Participants with incomplete information on education level, household income, smoking status, drinking status, and physical activity were further excluded. A total of 98,042 participants aged 18 years and older were included for the association between sociodemographic factors and metabolic syndrome prevalence. Among them, 32,300 participants (13,741 men and 18,559 women) completed the FFQ for the association between dietary intake and metabolic syndrome prevalence. The characteristics of the inclusion and exclusion subjects are shown in S1 Table. There exists significant difference in area, smoking status, physical activity level, and blood pressure between the inclusion and exclusion subjects.

This survey was approved by the Ethical Committee of the National Institute for Nutrition and Food Safety, Chinese Center for Disease Control and Prevention. All participants provided written informed consent.

### Data collection

Height, weight, and waist circumference were measured in the morning before breakfast. Height and waist circumference were accurate to 0.1 cm and weight was accurate to 0.1 kg. BMI was calculated as weight in kilograms divided by height in meters squared. Overweight and obesity were defined according to classifications for Asian populations; thus, a BMI between 24.0 and 28.0 kg/m^2^ is considered overweight, and a BMI ≥ 28.0 kg/m^2^ is considered obesity [14]. Blood pressure levels were measured three times after 5 minutes of rest in a seated position, and the set interval between measurements was 1 minute. The mean of the three measurements was used for analysis. Hypertension was defined as any of the following: systolic pressure ≥ 140 mmHg; diastolic pressure ≥ 90 mmHg; use of antihypertensive medications; or self-reported hypertension [15, 16]. Fasting plasma glucose, TG, and HDL-C were measured by the hexokinase G-6- PDH method, the GPO-HMMPS glycerol blanking method, and the direct determination method, respectively. All measurements were conducted with the Hitachi 7600 automated biochemical analyzer and all reagents were produced by Wako Pure Chemical, Ltd. Diabetes was diagnosed according to the American Diabetes Association criteria as any of the following [17]: self-report of a physician’s diagnosis of diabetes; fasting plasma glucose ≥ 7.0 mmol/L; oral glucose tolerance test (OGTT) 2-h plasma glucose ≥ 11.1 mmol/L; hemoglobin A1c ≥ 6.5%; or use of anti-diabetic medications.

### Definition of metabolic syndrome

The diagnosis of metabolic syndrome was based on the updated NCEP ATPIII criteria [2] and included three or more of the following: (1) abdominal obesity (defined according to guidelines for Chinese populations as waist circumference ≥ 90 cm in men or ≥ 85 cm in women [18]); (2) TG ≥ 1.69 mmol/L; (3) HDL-C cholesterol < 1.03 mmol/L in men or < 1.29 mmol/L in women; (4) systolic blood pressure ≥ 130 mmHg or diastolic blood pressure ≥ 85 mmHg or use of antihypertensive medications; and (5) fasting plasma glucose ≥ 5.6 mmol/L or use of anti-diabetic medications.

### Assessment of dietary intake

In the present study, dietary intake over the past year was assessed with a validated semiquantitative FFQ [19]. The FFQ includes 100 food items. Participants were asked the frequency and amount of each food consumed. Using China Food Composition data [20], we collapsed the 100 food items into 14 predefined food groups (S2 Table). According to the frequency of food intake, each food group was classified into tertiles (low, moderate, and high).

### Assessment of covariates

Information including education level, household income, smoking status, drinking status, and physical activity was obtained by trained investigators from face-to-face interviews. We classified education level into uneducated, primary school, junior school, high school, and college or above. Household income was divided into < 10,000, 10,000–30,000, and ≥ 30,000 yuan. Smoking status was classified into never, ever, and current smokers. Drinking status was categorized as non-drinkers, moderate alcohol consumption (with an alcohol intake of less than 175 g by men and 105 g by women per week), and excessive alcohol consumption (with an alcohol intake of more than 175 g by men and 105 g by women per week). Physical activity level (PAL) was calculated according to the recommendation of the Institute of Medicine (IOM) [21] and was divided into quartiles.

### Statistical analysis

Continuous variables with normal distribution were presented as means ± standard deviation (SD) and compared between groups using z test. Skewed distribution variables were presented as medians (interquartile ranges) and compared between groups using non-parametric statistical hypothesis test including Wilcoxon rank test and Kruskal Wallis test. Categorical variables were expressed as number (percentages) and compared by the chi-square test. The 2010–2012 CNNHS adopted a complex, multistage, probability sampling design. The standardized prevalence of metabolic syndrome was calculated using the weight coefficients to represent the overall Chinese adult population aged 18 years or older. Weight coefficients accommodated sampling weight and post stratification weight. Sampling weight was computed based on the study design. Post stratification weight was stratified by area, age, and sex, and harmonized the sample structure of the survey with that of the 2010 Chinese population census. PROC SURVEYMEANS and PROC SURVEYFREQ were used for the calculation of means and prevalence. PROC SURVEYLOGISTIC was used to calculate the odd ratios (ORs) and 95% confidence intervals (CIs) of metabolic syndrome prevalence. A 2-sided *P* value < 0.05 was used to determine statistical significance. Data cleaning and statistical analyses were performed using SAS version 9.2 (SAS Institute).

## Results

The characteristics of the participants by sex are shown in Table 1. There was a significant difference between the two groups in terms of education level, smoking status, drinking status, and physical activity level. Men were more likely than women to be older, with higher levels of waist circumference, TG, blood pressure, and fasting plasma glucose and lower levels of BMI and HDL-C. Men had a higher prevalence of hypertension (24.9%) than women (21.5%).

**Table 1:** Characteristics of participants according to sex (n = 98042).

	Men	Women	*P*-value
N (%)	42036 (50.11)	56006 (49.89)	
Age (years)	52.92 ± 14.56	51.33 ± 14.25	< 0.001
Area, n (%)			0.28
Urban	20146 (49.59)	28504 (50.64)	
Rural	21890 (50.41)	27502 (49.36)	
Education, n (%)			< 0.001
Uneducated	2969 (4.11)	9716 (12.87)	
Primary school	11954 (21.93)	17399 (26.43)	
Junior school	16431 (46.11)	17735 (38.59)	
High school	7253 (19.12)	7675 (14.68)	
College and above	3429 (8.74)	3481 (7.42)	
Income, n (%)			0.005
< 10000	21937 (52.31)	29090 (54.28)	
10000–30000	17115 (40.81)	22982 (39.29)	
> 30000	2984 (6.88)	3934 (6.43)	
Smoking, n (%)			< 0.001
Current smoker	22698 (54.68)	1823 (2.22)	
Former smoker	3201 (4.88)	706 (1.05)	
Never smoker	16137 (40.44)	53477 (96.73)	
Drinking, n (%)			< 0.001
Never drinker	19200 (43.02)	48394 (87.27)	
Moderate alcohol drinker	15315 (39.58)	6709 (11.53)	
Excessive alcohol drinker	7521 (17.41)	903 (1.20)	
Physical activity, n (%)			< 0.001
Low	12222 (30.43)	12245 (25.33)	
Moderate	7628 (16.12)	16436 (28.71)	
High	11230 (30.47)	13203 (21.90)	
Very high	10956 (22.99)	14122 (24.07)	
BMI, n (%)			0.24
Normal	23189 (57.36)	29930 (58.46)	
Overweight	14067 (30.36)	18649 (29.74)	
Obesity	4780 (12.28)	7427 (11.80)	
BMI (kg/m^2^)	23.54 (21.31, 25.96)	23.69 (21.38, 26.22)	< 0.001
Waist circumference (cm)	83.61 ± 10.40	80.17 ± 9.95	< 0.001
TG (mmol/L)	1.17 (0.80, 1.80)	1.12 (0.78, 1.67)	< 0.001
HDL-C (mmol/L)	1.15 ± 0.34	1.22 ± 0.32	< 0.001
Systolic blood pressure (mmHg)	126.98 ± 19.77	124.35 ± 21.53	< 0.001
Diastolic blood pressure (mmHg)	80.06 ± 11.69	77.70 ± 11.72	< 0.001
Fasting plasma glucose (mmol/L)	5.21 (4.74, 5.75)	5.17 (4.72, 5.68)	< 0.001
Abdominal obesity, n (%)	12223 (27.06)	17799 (26.62)	0.53
Diabetes, n (%)	4561 (6.79)	5599 (6.62)	0.49
Hypertension, n (%)	14769 (24.93)	17517 (21.52)	< 0.001

Data are mean ± standard deviation for normally distributed or medians (interquartile ranges) for skewed parameters, or number (%).

Abbreviations: BMI, body mass index; TG, triglyceride; HDL-C, high-density lipoprotein cholesterol.

The characteristics of the participants according to metabolic syndrome status are shown in Table 2. The individuals with metabolic syndrome were more likely than the normal participants to be older, with higher levels of BMI, waist circumference, TG, blood pressure, and fasting plasma glucose, and lower levels of HDL-C. The metabolic syndrome prevalence was higher among North and East residents, but lower among North-West, South-West and North-East residents than among the general populations. Participants with metabolic syndrome were more likely to have obesity (33.6%), abdominal obesity (72.0%), diabetes (18.7%), and hypertension (52.2%).

**Table 2:** Characteristics of participants according to metabolic syndrome status (n = 98042).

Variables	Normal	Metabolic syndrome	*P*-value
N (%)	67451 (75.80)	30591 (24.20)	
Men, n (%)	29665 (49.82)	12371 (50.99)	0.15
Age (years)	49.99 ± 14.76	56.45 ± 12.50	< 0.001
Area, n (%)			< 0.001
Urban	31307 (48.30)	17343 (55.80)	
Rural	36144 (51.70)	13248 (44.20)	
Smoking, n (%)			< 0.001
Current smoker	17820 (28.85)	6701 (27.45)	
Former smoker	2346 (2.67)	1561 (3.90)	
Never smoker	47285 (68.48)	22329 (68.65)	
Drinking, n (%)			< 0.001
Never drinker	45696 (64.88)	21898 (65.80)	
Moderate alcohol drinker	15784 (26.15)	6240 (23.80)	
Excessive alcohol drinker	5971 (8.97)	2453 (10.40)	
Physical activity level, n (%)			< 0.001
Low	15956 (27.54)	8511 (28.96)	
Moderate	15572 (21.69)	8492 (24.64)	
High	16518 (26.04)	7915 (26.67)	
Very high	19405 (24.74)	5673 (19.73)	
Region			< 0.001
North	9638 (67.10)	6310 (32.90)	
East	5581 (71.09)	3200 (28.91)	
South-Central	18432 (75.53)	8264 (24.47)	
North-West	15434 (76.93)	6774 (23.07)	
South-West	10605 (82.47)	3043 (17.53)	
North-East	7761 (81.93)	3000 (18.07)	
BMI (kg/m^2^)	22.55 (20.66, 24.57)	26.30 (24.24, 30.59)	< 0.001
Waist circumference (cm)	77.99 ± 8.62	89.70 ± 9.01	< 0.001
TG (mmol/L)	0.96 (0.70, 1.31)	1.89 (1.31, 2.62)	< 0.001
HDL-C (mmol/L)	1.28 ± 0.32	0.99 ± 0.25	< 0.001
Systolic blood pressure (mmHg)	120.60 ± 19.15	136.22 ± 20.37	< 0.001
Diastolic blood pressure (mmHg)	76.26 ± 10.93	84.13 ± 11.72	< 0.001
Fasting plasma glucose (mmol/L)	5.03 (4.62, 5.43)	5.70 (5.12, 6.33)	< 0.001
Obesity, n (%)	3241 (5.15)	8966 (33.61)	< 0.001
Abdominal obesity, n (%)	8631 (12.41)	21391 (72.01)	< 0.001
Diabetes, n (%)	2914 (2.88)	7246 (18.69)	< 0.001
Hypertension, n (%)	13894 (13.99)	18392 (52.16)	< 0.001

Data are mean ± standard deviation for normally distributed or medians (interquartile ranges) for skewed parameters, or number (%).

Abbreviations: BMI, body mass index; TG, triglyceride; HDL-C, high-density lipoprotein cholesterol.

The overall metabolic syndrome prevalence and its risk factors are shown in Table 3. Participants living in urban areas (27.0%) had a higher metabolic syndrome prevalence than rural residents (21.5%). The prevalence of metabolic syndrome was positively associated with age and household income, but negatively associated with physical activity level (*P* for trend < 0.05). Individuals with overweight (OR: 6.40; 95% CI: 5.90–6.94) or obesity (OR: 25.04; 95% CI: 22.27–28.15) had a higher metabolic syndrome prevalence than normal individuals. The prevalence of metabolic syndrome was relatively lower among participants living in the South-Central, South-West, and North-East regions (OR = 0.73, 0.61, and 0.77, respectively) than among North residents. Significantly interactions were found for sex and age, area, education, income, drinking status, physical activity level, and BMI with metabolic syndrome prevalence (all *P*-interaction < 0.01).

**Table 3:** The metabolic syndrome prevalence and its risk factors in total sample (n = 98042).

	N	Prevalence	OR (95% CI)	*P*-value	*P*-interaction ^a^
**Age, years**					< 0.001
18–44	30887	16.03	1.00 (ref)		
45–54	23264	32.12	2.27 (2.08–2.47)	< 0.001	
55–64	25046	36.97	3.16 (2.88–3.48)	< 0.001	
≥ 65	18845	37.81	4.02 (3.60–4.48)	< 0.001	
*P*-trend			< 0.001		
**Area**					< 0.001
Urban	48650	26.95	1.00 (ref)		
Rural	49392	21.45	0.92 (0.82–1.04)	0.19	
**Education**					< 0.001
Uneducated	12685	31.42	1.00 (ref)		
Primary school	29353	26.27	0.91 (0.83–1.01)	0.07	
Junior school	34166	23.00	0.89 (0.79–0.99)	0.03	
High school	14928	22.55	0.76 (0.68–0.86)	< 0.001	
College and above	6910	20.23	0.78 (0.64–0.94)	0.01	
*P*-trend			0.07		
**Income, n (%)**					< 0.001
< 10000	51027	23.31	1.00 (ref)		
10000–30000	40097	25.17	1.08 (0.99–1.18)	0.07	
> 30000	6918	25.53	1.19 (1.05–1.35)	0.007	
*P*-trend			0.005		
**Smoking, n (%)**					0.05
Never smoker	24521	24.25	1.00 (ref)		
Current smoker	3907	23.30	1.02 (0.95–1.10)	0.59	
Former smoker	69614	31.82	1.04 (0.92–1.18)	0.49	
**Drinking, n (%)**					0.01
Never drinker	67594	24.46	1.00 (ref)		
Moderate alcohol drinker	22024	22.52	0.96 (0.89–1.04)	0.29	
Excessive alcohol drinker	8424	27.01	0.99 (0.90–1.10)	0.85	
**Physical activity, n (%)**					< 0.001
Low	24467	25.14	1.00 (ref)		
Moderate	24064	26.62	1.01 (0.92–1.10)	0.89	
High	24433	24.65	0.90 (0.83–0.98)	0.02	
Very high	25078	20.30	0.79 (0.72–0.86)	< 0.001	
*P*-trend			< 0.001		
**BMI, kg/m**^**2**^					< 0.001
Normal	53119	8.31	1.00 (ref)		
Overweight	32716	37.45	6.40 (5.90–6.94)	< 0.001	
Obesity	12207	67.57	25.04 (22.27–28.15)	< 0.001	
*P*-trend			< 0.001		
**Region**					0.24
North	15948	32.90	1.00 (ref)		
East	8781	28.91	0.76 (0.55–1.06)	0.11	
South-Central	26696	24.47	0.73 (0.61–0.87)	< 0.001	
North-West	22208	23.07	0.89 (0.74–1.08)	0.23	
South-West	13648	17.53	0.61 (0.51–0.72)	< 0.001	
North-East	10761	18.07	0.77 (0.64–0.92)	0.005	

Abbreviations: BMI, body mass index; OR, odd ratio; CI, confidence interval.

^a^: Interactions between sex and stratified factors. Adjusted for gender, age, area, education level, Household income, smoking status, drinking status, physical activity level, BMI, and region.

Table 4 shows the metabolic syndrome prevalence and its risk factors according to sex. The prevalence of metabolic syndrome was 24.6% in men, and 23.8% in women. The prevalence of metabolic syndrome was positively associated with age in men and women. Among men, metabolic syndrome prevalence was negatively associated with the physical activity level (*P* for trend < 0.001). Metabolic syndrome prevalence was inversely associated with the education level among women (*P* for trend < 0.001). Individuals with obesity had a higher metabolic syndrome prevalence among men and women (OR = 28.24, 20.40, respectively) than normal participants.

**Table 4:** The metabolic syndrome prevalence and its risk factors according to sex (n = 98042).

	Men (n = 42036)			Women (n = 56006)		
	N	Prevalence	OR (95% CI) ^a^	N	Prevalence	OR (95% CI) ^a^
**Age, years**						
18–44	12419	20.56	1.00 (ref)	18468	11.41	1.00 (ref)
45–54	9573	31.41	1.83 (1.62–2.06) ***	13691	32.83	3.02 (2.74–3.32) ***
55–64	10951	29.68	1.87 (1.62–2.15) ***	14095	44.34	5.60 (4.90–6.39) ***
≥ 65	9093	28.48	2.23 (1.94–2.56) ***	9752	46.36	7.65 (6.61–8.85) ***
*P*-trend			< 0.001			< 0.001
**Area**						
Urban	20146	28.63	1.00 (ref)	28504	25.29	1.00 (ref)
Rural	21890	20.70	0.92 (0.79–1.05)	27502	22.22	0.94 (0.83–1.07)
**Education**						
Uneducated	2969	18.92	1.00 (ref)	9716	35.44	1.00 (ref)
Primary school	11954	21.56	1.13 (0.98–1.31)	17399	30.20	1.11 (1.00–1.23) *
Junior school	16431	24.58	1.23 (1.04–1.45) *	17735	21.10	1.03 (0.91–1.17)
High school	7253	27.86	1.21 (1.00–1.47) *	7675	15.60	0.67 (0.56–0.80) ***
College and above	3429	28.25	1.16 (0.86–1.56)	3481	10.76	0.70 (0.55–0.90) **
*P*-trend			0.09			0.006
**Income, n (%)**						
< 10000	21937	22.02	1.00 (ref)	29090	24.57	1.00 (ref)
10000–30000	17115	27.07	1.11 (1.00–1.23)	22982	23.18	1.01 (0.91–1.12)
> 30000	2984	30.03	1.19 (0.99–1.42)	3934	20.70	1.10 (0.92–1.31)
*P*-trend			0.03			0.39
**Smoking, n (%)**						
Never smoker	16137	25.89	1.00 (ref)	53477	23.56	1.00 (ref)
Current smoker	22698	22.98	1.03 (0.94–1.13)	1823	31.18	1.12 (0.93–1.35)
Former smoker	3201	32.72	1.19 (1.02–1.39) *	706	27.61	0.98 (0.73–1.32)
**Drinking, n (%)**						
Never drinker	19200	24.81	1.00 (ref)	48394	24.29	1.00 (ref)
Moderate alcohol drinker	15315	23.32	0.90 (0.82–0.99) *	6709	19.76	0.95 (0.85–1.06)
Excessive alcohol drinker	7521	27.18	1.00 (0.89–1.11)	903	24.51	0.73 (0.52–1.02)
**Physical activity, n (%)**						
Low	12222	27.49	1.00 (ref)	12245	22.31	1.00 (ref)
Moderate	7628	31.43	1.08 (0.96–1.22)	16436	23.91	1.02 (0.91–1.14)
High	11230	23.65	0.80 (0.72–0.88) ***	13203	26.04	1.02 (0.88–1.17)
Very high	10956	17.39	0.70 (0.62–0.79) ***	14122	23.09	0.93 (0.83–1.06)
*P*-trend			< 0.001			0.32
**BMI, kg/m**^**2**^						
Normal	23189	7.52	1.00 (ref)	29930	9.10	1.00 (ref)
Overweight	14067	38.90	7.40 (6.72–8.14) ***	18649	35.96	5.26 (4.71–5.86) ***
Obesity	4780	69.32	28.24 (24.03–33.19) ***	7427	65.75	20.40 (17.98–23.16) ***
*P*-trend			< 0.001			< 0.001
**Region**						
North	6967	34.20	1.00 (ref)	8981	31.54	1.00 (ref)
East	3719	29.52	0.79 (0.56–1.11)	5062	28.30	0.76 (0.54–1.06)
South-Central	11699	25.48	0.79 (0.66–0.95) *	14997	23.43	0.68 (0.55–0.83) **
North-West	9364	23.22	0.88 (0.73–1.06)	12844	22.92	0.89 (0.72–1.10)
South-West	5740	15.63	0.62 (0.51–0.76) ***	7908	19.36	0.59 (0.48–0.72) ***
North-East	4547	18.86	0.88 (0.72–1.08)	6214	17.31	0.68 (0.56–0.84) ***

Abbreviations: BMI, body mass index; OR, odd ratio; CI, confidence interval.

^a^: Adjusted for age, area, education level, Household income, smoking status, drinking status, physical activity level, BMI, and region. Except the variable of interest.

*: *P* < 0.05;

**: *P* < 0.01;

***: *P* < 0.001

**Fig 1:**
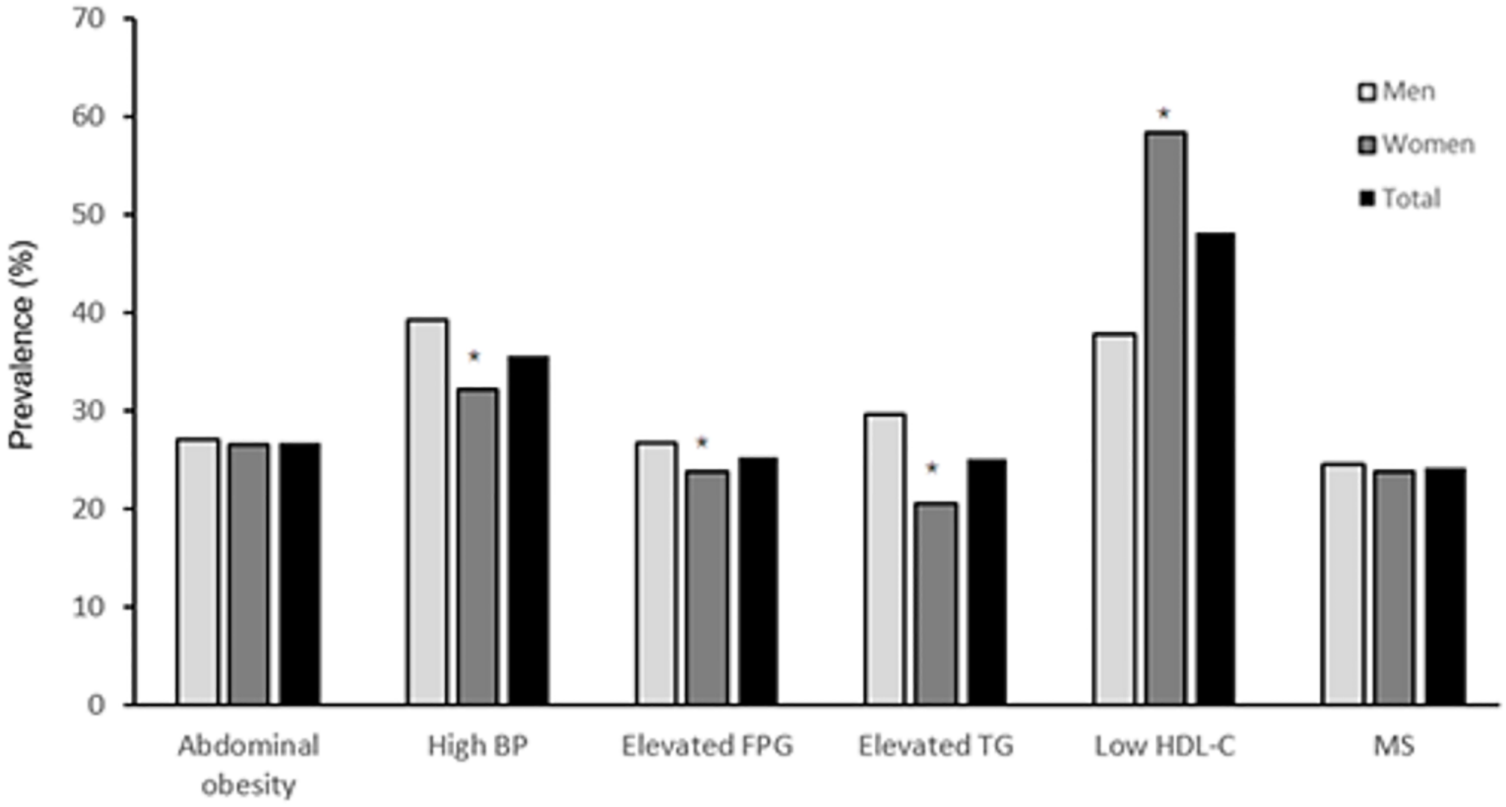
Sex disparity prevalence of metabolic components among adults in China. BP, blood pressure; FPG, fasting plasma glucose; HDL-C, high-density lipoprotein cholesterol. *: *P* < 0.01 for men: women difference in prevalence.

**Fig 2:**
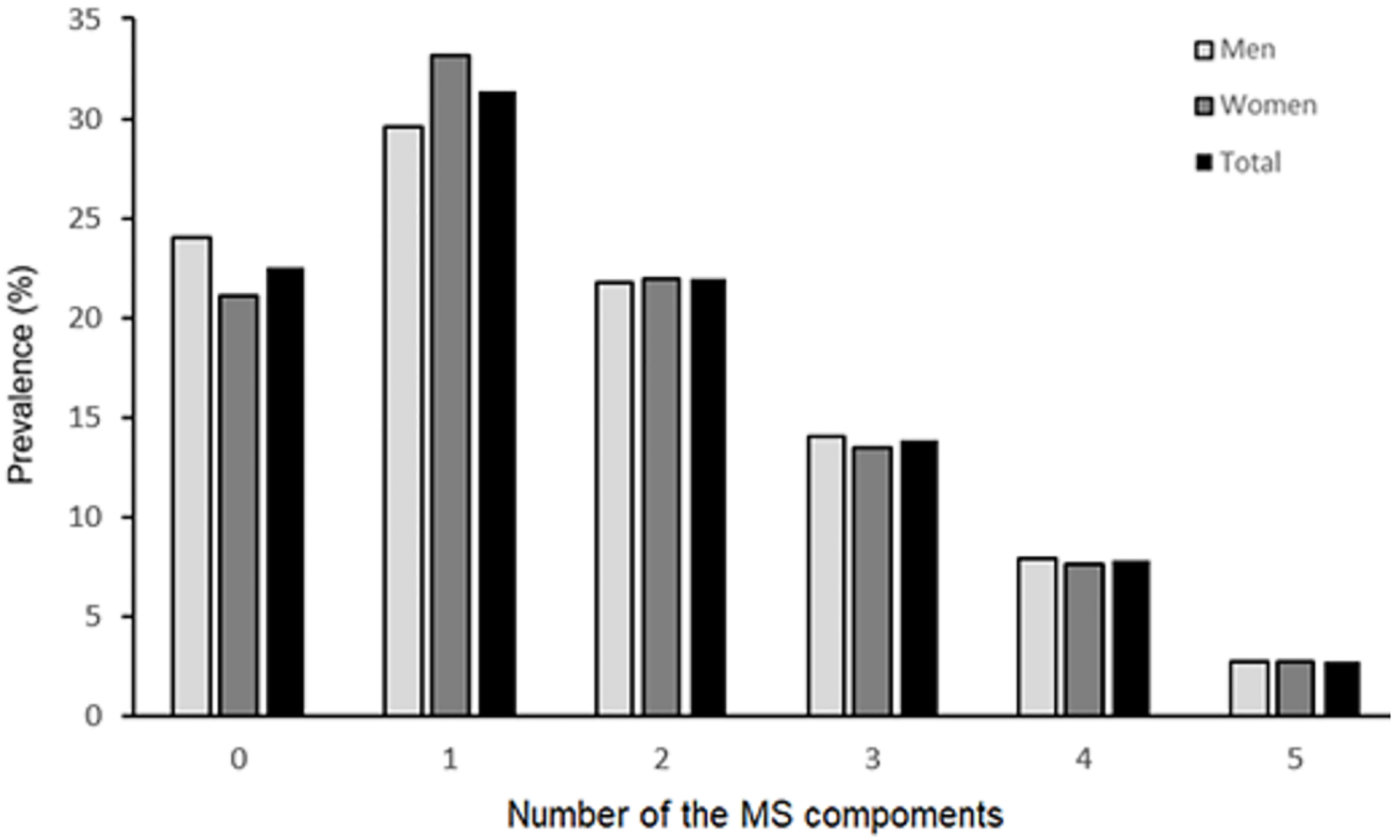
Sex disparity prevalence of metabolic syndrome and number of metabolic components among adults in China. MS, metabolic syndrome.

The prevalence of metabolic components is shown in Fig 1. Women had a higher prevalence of lower HDL-C (58.3%) than men (37.8%), whereas men had a higher prevalence of high blood pressure (39.2%), elevated fasting plasma glucose (26.7%), and elevated TG (29.6%) than women (32.1%, 23.8%, and 20.5%, respectively). The prevalence of abdominal obesity and metabolic syndrome was not significantly different between men and women (*P* > 0.05). Fig 2 shows the prevalence of one or more metabolic components. Overall, 31.4% (29.6% in men and 33.2% in women) of the participants had one metabolic component, and 24.2% of the participants (24.6% of men, and 23.8% of women) had three or more metabolic components, which is the definition of metabolic syndrome.

Table 5 shows the frequency of food intake of the participants. Men consumed more pork and organ meats and had a lower intake of fungi and algae, fruit, and dairy products than women.

**Table 5:** Frequency of foods intake of participants according to sex.

Food (times/week)	Total (n = 32300)	Men (n = 13741)	Women (n = 18559)
Rice and rice products	14.00 (5.00, 14.00)	14.00 (5.00, 16.00)	14.00 (5.00, 14.00)
Wheat and products	7.00 (2.00, 10.00)	7.00 (2.00, 10.08)	7.00 (2.00, 10.00)
Starchy tubers	1.00 (0.50, 3.00)	1.00 (0.50, 3.00)	1.00 (0.50, 3.00)
Soybean products	2.75 (1.12, 5.50)	2.75 (1.10, 5.31)	2.75 (1.12, 5.50)
Vegetables	15.00 (9.50, 22.00)	15.00 (9.50, 22.00)	15.00 (9.50, 22.00)
Fungi and algae	1.12 (0.50, 2.50)	1.08 (0.50, 2.50) *	1.15 (0.50, 2.50)
Fruit	4.00 (2.00, 7.25)	3.75 (1.85, 7.00) *	4.13 (2.00, 7.62)
Dairy products	2.00 (0.75, 7.00)	2.00 (0.58, 7.00) *	2.29 (0.75, 7.00)
Pork	3.00 (1.08, 7.00)	3.02 (1.25, 7.00) *	3.00 (1.03, 7.00)
Poultry	0.50 (0.25, 1.06)	0.50 (0.25, 1.06)	0.50 (0.25, 1.08)
Organ meats	0.25 (0.10, 0.58)	0.26 (0.10, 0.62) *	0.25 (0.10, 0.54)
Fish	1.12 (0.42, 2.75)	1.12 (0.44, 2.75)	1.12 (0.40, 2.79)
Eggs	3.00 (1.50, 7.00)	3.00 (1.44, 7.00)	3.00 (1.50, 7.00)
Nuts	0.83 (0.31, 2.00)	0.83 (0.29, 2.00)	0.83 (0.31, 2.00)

Data are medians (interquartile ranges).

*: Compared with women, *P* < 0.05

Associations between the frequency of foods intake and metabolic syndrome prevalence among men are shown in Table 6. A high frequency of fungi and algae intake was associated with an increased metabolic syndrome prevalence after adjustment for confounding factors. Associations between food intake and metabolic syndrome prevalence among women are shown in Table 7. In contrase to the first tertile of pork and nuts, the ORs (95% CIs) of metabolic syndrome prevalence for the highest tertile were 0.87 (0.78–0.95), and 0.88 (0.78–0.98), respectively.

**Table 6:** Associations between the frequency of foods intake and metabolic syndrome prevalence in men (n = 13741).

	Times/week			*P*-trend ^a^
	Low	Moderate	High	
Food				
Rice and rice products	1.00 (ref)	0.98 (0.85–1.13)	0.96 (0.86–1.06)	0.77
Wheat and products	1.00 (ref)	1.03 (0.92–1.16)	1.01 (0.90–1.14)	0.38
Starchy tubers	1.00 (ref)	0.94 (0.82–1.07)	0.96 (0.86–1.08)	0.57
Soybean products	1.00 (ref)	1.08 (0.97–1.21)	0.98 (0.87–1.11)	0.86
Vegetables	1.00 (ref)	1.09 (0.98–1.22)	1.08 (0.97–1.21)	0.16
Fungi and algae	1.00 (ref)	1.04 (0.92–1.17)	1.24 (1.09–1.40)	< 0.001
Fruits	1.00 (ref)	0.97 (0.87–1.09)	0.93 (0.83–1.05)	0.34
Dairy products	1.00 (ref)	0.90 (0.76–1.06)	0.99 (0.83–1.17)	0.95
Pork	1.00 (ref)	0.93 (0.83–1.04)	0.89 (0.79–1.00)	0.05
Poultry	1.00 (ref)	0.99 (0.87–1.12)	1.07 (0.95–1.21)	0.19
Organ meats	1.00 (ref)	0.99 (0.83–1.17)	1.05 (0.90–1.24)	0.54
Fish	1.00 (ref)	0.99 (0.88–1.12)	1.06 (0.94–1.20)	0.24
Eggs	1.00 (ref)	1.00 (0.89–1.13)	0.99 (0.87–1.11)	0.99
Nuts	1.00 (ref)	0.97 (0.85–1.10)	1.02 (0.89–1.16)	0.74

BMI, body mass index.

^a^: Adjusted for the age, area, education, income, smoking status, drinking status, physical activity level, and BMI.

**Table 7:** Associations between the frequency of foods intake and metabolic syndrome prevalence in women (n = 18559).

	Times/week			*P*-trend ^a^
	Low	Moderate	High	
Rice and rice products	1.00 (ref)	0.96 (0.86–1.08)	0.93 (0.85–1.02)	0.82
Wheat and products	1.00 (ref)	1.01 (0.92–1.11)	1.02 (0.93–1.13)	0.23
Starchy tubers	1.00 (ref)	0.94 (0.84–1.04)	0.94 (0.85–1.04)	0.18
Soybean products	1.00 (ref)	0.94 (0.85–1.03)	1.02 (0.92–1.12)	0.61
Vegetables	1.00 (ref)	1.03 (0.94–1.12)	1.06 (0.96–1.16)	0.22
Fungi and algae	1.00 (ref)	1.04 (0.94–1.15)	1.01 (0.90–1.12)	0.85
Fruits	1.00 (ref)	0.94 (0.86–1.03)	0.91 (0.82–1.00)	0.07
Dairy products	1.00 (ref)	0.89 (0.77–1.02)	0.91 (0.79–1.06)	0.19
Pork	1.00 (ref)	0.94 (0.86–1.03)	0.87 (0.78–0.95)	0.007
Poultry	1.00 (ref)	0.95 (0.85–1.06)	1.09 (0.98–1.22)	0.09
Organ meats	1.00 (ref)	1.09 (0.94–1.26)	1.03 (0.89–1.19)	0.71
Fish	1.00 (ref)	0.94 (0.85–1.04)	1.04 (0.94–1.15)	0.39
Eggs	1.00 (ref)	0.98 (0.89–1.08)	0.93 (0.84–1.03)	0.14
Nuts	1.00 (ref)	0.92 (0.83–1.03)	0.88 (0.78–0.98)	0.02

BMI, body mass index.

^a^: Adjusted for the age, area, education, income, smoking status, drinking status, physical activity level, and BMI.

## Discussion

In the present study, we investigated different associations by sex between metabolic syndrome prevalence and its associated factors among a Chinese population. Based on the NCEP ATP III-modified criteria, our results showed that the overall prevalence of metabolic syndrome was 24.2% (24.6% in men and 23.8% in women) among Chinese adults. Our results also suggested that the metabolic syndrome prevalence was positively associated with age. The metabolic syndrome prevalence was negatively associated with the physical activity level in men but inversely associated with the education level in women. The frequent consumption of fungi and algae was an underlying risk factor for metabolic syndrome in men, whereas the frequent consumption of nuts and pork was associated with a decreased metabolic syndrome prevalence in women.

Over the past decades, the metabolic syndrome prevalence has increased markedly worldwide [3, 5, 22], which may be explained by urbanization, an aging population, lifestyle change, and nutritional transition. Data from the National Health and Nutrition Examination Survey reported that the prevalence of metabolic syndrome among American populations aged 20 years and older increased from 32.9% in 2003–2004 to 34.7% in 2011–2012 [3]. A study conducted in urban Eastern India among adults aged 20–80 years demonstrated that the aged-standardized prevalence of metabolic syndrome was 33.5% overall: 24.9% in men and 42.3% in women [5]. The Dongfeng-Tongji Cohort study conducted in Wuhan reported that the overall metabolic syndrome prevalence was 33.2% among middle-aged and elderly Chinese populations [23]. The findings in the present study and previous surveys indicate that metabolic syndrome has become a serious public health problem and highlights the urgent need to prevent and treat metabolic syndrome in China and other populations.

The standardized prevalence of metabolic syndrome in the present study was lower than that reported in some previous studies [7, 9, 11]. The difference in prevalence is mainly explained by the definition of metabolic syndrome and the selection of study participants. The 2010 China Noncommunicable Disease Surveillance, which involvd 97,098 participants aged 18 years and older, reported that the prevalence of metabolic syndrome was 33.9% (31.0% in men and 36.8% in women) using NCEP ATP III criteria [7]. In 2012, a cross-sectional study conducted among 11,496 Chinese participants aged 35 years and older reported that metabolic syndrome prevalence was 39.0% overall and 31.4% in men and 45.6% in women, all by NCEP ATP III criteria [11]. For these studies, the cutoff for abdominal obesity was waist circumference ≥ 90 cm in men or ≥ 80 cm in women, according to the ethnic criteria for Asians [2]. The National Diabetes and Metabolic Disorders Survey conducted in 2007–2008 among 45,172 Chinese adults aged 20 years and older suggested that the prevalence of metabolic syndrome was 21.9% (25.8% in men and 18.0% in women) using the cutoffs recommended by the Chinese Joint Committee for Developing Chinese Guidelines (JCDCG) [24]; these findings were consistent with our results. According to the JCDCG criteria, abdominal obesity was defined as waist circumference ≥ 90 cm in men or ≥ 85 cm in women.

Previous studies showed that the prevalence of metabolic syndrome was higher in women than in men [3, 7]. However, our results found that the prevalence of metabolic syndrome in men (24.6%) was not significantly different from that in women (23.8%), which was consistent with other studies [9, 25, 26]. The following reasons may contribute to the sex differences in the distribution of metabolic syndrome. First, postmenopausal status is associated with an increased risk of central obesity and insulin resistance [27], which might account for the different metabolic syndrome prevalence between men and women. Second, our findings demonstrated that obesity was significantly associated with an increased risk of metabolic syndrome. A previous study indicated that pregnant women had a higher prevalence of obesity and that the more times a woman becomes pregnant, the more likely the woman is to be obesity, thus, increasing the risk of having metabolic syndrome [28].

Our results also suggested that there exist different associations of risk factors with metabolic syndrome prevalence among men and women. As shown in Table 4, with a higher level of education, the prevalence of metabolic syndrome was higher in men, but lower in women. A possible explanation for this result is that men with a higher education level and household income may spend more time sitting in the office, may have little time to exercise, may frequently consume high-fat foods, and may suffer from work-related mental health problems. Further studies are needed to investigate the potential mechanisms of these results.

The present study also suggested that the frequent consumption of pork was associated with a decreased metabolic syndrome prevalence. The possible explanation for this association is that participants with metabolic syndrome may change their lifestyles and dietary patterns, which may lead to this result. In addition, participants who frequently consumed pork also had a higher intake of vegetables, fruits, dairy products, and fish. Several prospective studies demonstrated that food groups including vegetables, fruits, dairy products, and fish were inversely associated with metabolic syndrome prevalence [29–32]. Further longitudinal surveys are needed to interpret the observed associations in terms of the cause and effect.

This study was based on data from the CHNNS, which was conducted among participants from all 31 provinces, autonomous regions, and municipalities using a complex, multistage, probability sampling design, therefore providing this study with good representativeness; its findings may be seen as convincing. However, several limitations should be considered. First, the cross-sectional design limits the ability to address causal relationships between risk factors and metabolic syndrome. Second, the prevalence of metabolic syndrome was based on a single assessment of blood samples, which may lead to minor inaccuracies. Third, because the sociodemographic characteristics and dietary information were obtained through a questionnaire, this may lead to recall bias. Fourth, the characteristics of subjects included in dietary food intake assessment differed from those of excluded subjects, which may bias our results.

## Conclusion

In conclusion, the present study shows that the prevalence of metabolic syndrome in men was not significantly different from that in women. Our results also suggest sex-specific associations between multiple risk factors including sociodemographic characteristics, lifestyle, and nutrition intake, with metabolic syndrome. Our findings indicate that metabolic syndrome has become a serious public health problem, and thus, a need exists to develop strategies aimed at the prevention and treatment of metabolic syndrome in China.

## Supporting information

S1 Table Characteristics of inclusion and exclusion subjects. (DOCX)

S2 Table Food items in the food groups. (DOCX)

S3 Table Analysis data set. (XLSX)
