# Supplementary material for: Metabolic syndrome prevalence and its risk factors among adults in China: A nationally representative cross-sectional study
Source: PLoS One. 2018 Jun 19;13(6):e0199293. doi: 10.1371/journal.pone.0199293 (PMC6007893; doi:10.1371/journal.pone.0199293)
Supplement: S1 Table — (DOCX) [file pone.0199293.s001.docx]

**Supplementary Table 1 Characteristics of inclusion and exclusion subjects**

| **Variables** | **Exclusion subjects** | **Inclusion subjects** | ***P*-value** |
| --- | --- | --- | --- |
| N (%) | 65742 (67.05) | 32300 (32.95) |  |
| Men, n (%) | 28295 (43.04) | 13741 (42.54) | 0.14 |
| Age (years) | 51.99 ± 14.50 | 52.06 ± 14.22 | 0.47 |
| Area, n (%) |  |  | 0.007 |
| Urban | 32820 (49.92) | 15830 (49.01) |  |
| Rural | 32922 (50.08) | 16470 (50.99) |  |
| Smoking, n (%) |  |  | 0.002 |
| Current smoker | 16572 (25.21) | 7949 (24.61) |  |
| Former smoker | 2698 (4.10) | 1209 (3.74) |  |
| Never smoker | 46472 (70.69) | 23142 (71.65) |  |
| Drinking, n (%) |  |  | 0.64 |
| Never drinker | 45359 (69) | 22235 (68.84) |  |
| Moderate alcohol drinker | 14773 (22.47) | 7251 (22.45) |  |
| Excessive alcohol drinker | 5610 (8.53) | 2814 (8.71) |  |
| Physical activity level, n (%) |  |  | 0.003 |
| Low | 16396 (24.94) | 8071 (24.99) |  |
| Moderate | 16273 (24.75) | 7791 (24.12) |  |
| High | 16476 (25.06) | 7957 (24.63) |  |
| Very high | 16597 (25.25) | 8481 (26.26) |  |
| BMI (kg/m^2^) | 23.88 ± 3.58 | 23.90 ± 3.55 | 0.44 |
| Waist circumference (cm) | 81.64 ± 10.30 | 81.65 ± 10.28 | 0.84 |
| TG (mmol/L) | 1.43 ± 1.04 | 1.44 ± 1.05 | 0.17 |
| HDL-C (mmol/L) | 1.19 ± 0.33 | 1.19 ± 0.33 | 0.66 |
| Systolic blood pressure (mmHg) | 125.35 ± 20.86 | 125.74 ± 20.77 | 0.006 |
| Diastolic blood pressure (mmHg) | 78.63 ± 11.76 | 78.88 ± 11.76 | 0.001 |
| Fasting plasma glucose (mmol/L) | 5.39 ± 1.36 | 5.40 ± 1.37 | 0.08 |
| Obesity, n (%) | 8180 (12.44) | 4027 (12.47) | 0.39 |
| Abdominal obesity, n (%) | 20150 (30.65) | 9872 (30.56) | 0.78 |
| Diabetes, n (%) | 6756 (10.28) | 3404 (10.54) | 0.21 |
| Hypertension, n (%) | 21673 (32.97) | 10613 (32.86) | 0.73 |
