# Supplementary material for: Metabolic syndrome prevalence and its risk factors among adults in China: A nationally representative cross-sectional study
Source: PLoS One. 2018 Jun 19;13(6):e0199293. doi: 10.1371/journal.pone.0199293 (PMC6007893; doi:10.1371/journal.pone.0199293)
Supplement: S2 Table — (DOCX) [file pone.0199293.s002.docx]

**Supplementary Table 2 Food items in the food groups**

| **Food groups** | **Foods included in the group** |
| --- | --- |
| Rice and rice products | Round-grained rice, long-grained rice, glutinous rice |
| Wheat and products | Wheat bun, wheat noodles |
| Starchy tubers | Potato, sweet potato |
| Soybean products | Soybeans, and products |
| Vegetables | Cabbage, eggplant, carrot, pepper, lettuce, rape, tomato, cauliflower |
| Fungi and algae | Mushroom, kelp, laver |
| Fruits | Apple, pear, peach, date, grape, watermelon, orange, other fruit |
| Dairy products | Milk and products |
| Pork | Pork and pork products |
| Poultry | Chicken, duck, goose |
| Organ meats | Organ meats |
| Aquatic products | Fish, shrimp, crab, shellfish |
| Eggs | Eggs |
| Nuts | Nuts |
